# Challenges and future of biomarker tests in the era of precision oncology: Can we rely on immunohistochemistry (IHC) or fluorescence *in situ* hybridization (FISH) to select the optimal patients for matched therapy?

**DOI:** 10.18632/oncotarget.19809

**Published:** 2017-08-01

**Authors:** Young Kwang Chae, Ayush Arya, Lauren Chiec, Hiral Shah, Ari Rosenberg, Sandip Patel, Kirtee Raparia, Jaehyuk Choi, Derek A. Wainwright, Victoria Villaflor, Massimo Cristofanilli, Francis Giles

**Affiliations:** ^1^ Developmental Therapeutics Program of the Division of Hematology Oncology, Chicago, IL, USA; ^2^ Robert H. Lurie Comprehensive Cancer Center of Northwestern University, Chicago, IL, USA; ^3^ Northwestern University Feinberg School of Medicine, Chicago, IL, USA; ^4^ University of California San Diego, San Diego, CA, USA

**Keywords:** IHC, FISH, predictive, biomarker, targeted therapy

## Abstract

Molecular techniques have improved our understanding of the pathogenesis of cancer development. These techniques have also fueled the rational development of targeted drugs for patient populations stratified by their genetic characteristics. These novel methods have changed the classic paradigm of diagnostic pathology; among them are IHC, FISH, polymerase chain reaction (PCR) and microarray technology. IHC and FISH detection methods for human epidermal growth factor receptor-2 (HER2), epidermal growth factor receptor (EGFR) and programmed death ligand-1 (PD-L1) were recently approved by the Food and Drug Administration (FDA) as routine clinical practice for cancer patients. Here, we discuss general challenges related to the predictive power of these molecular biomarkers for targeted therapy in cancer medicine. We will also discuss the prospects of utilizing new biomarkers for fibroblast growth factor receptor (FGFR) and hepatocyte growth factor receptor (cMET/MET) targeted therapies for developing new and robust predictive biomarkers in oncology.

## Introduction to IHC and FISH

The advent of modern laboratory techniques have been instrumental in shaping medicine. These techniques serve as the means to acquire an in-depth knowledge of numerous pathological processes, and have expanded the role of medical laboratories beyond simple diagnostics. It is now possible to obtain reliable prognostic information for several disease processes. Furthermore, the outcome of treatment with particular therapeutic regimens can often be predicted with some certainty.

The concept of IHC was first introduced by Coons in 1941 [1]. This technique involves a multi-stage procedure for tissue fixation followed by interpretation of findings [2]. It detects specific antigens in histologic specimens with the use of target-specific antibodies coupled with colorimetric or fluorescent reagents, facilitating anatomical localization of proteins and phosphoproteins, *in situ* [3].

IHC is an important ancillary technique that is used for accomplishing a wide range of goals in modern laboratories including the studying the pathogenesis of several disease processes [4, 5]. It is frequently used to characterize many types of neoplasms, thus providing a histologic diagnosis and appropriate subtype of tumor. It can also assist in distinguishing between benign and malignant cell populations for certain tissues. Furthermore, it is used for determining the distribution and differential expression of particular biomarkers in a tissue specimen. This in-turn provides vital information for guiding the decision making process in terms of patient treatment [6–9].

*In situ* hybridization (ISH) was first described in the year 1969 by Joseph G. Gall [10]. Fluorescent ISH (FISH) is a variant of ISH that utilizes DNA probes that anneal with target gene sequences [11]. The DNA probes are labeled with fluorescent markers that allow detection of hybridization through fluorescence microscopy. This permits the identification of specific genomic aberrations in the sample DNA [11]. RNA *in situ* hybridization (RNA ISH) is another variant of ISH that is used for the detection of RNA sequences of interest [12, 13].

ISH is a fundamental technique that is commonly used for diagnostic and research purposes while FISH is routinely used to diagnose genetic diseases. FISH also serves an important role in diverse research domains such as documentation of novel oncogenes and gene mapping [11]. RNA ISH has enabled the detection of intracellular molecules such as messenger-RNA (mRNA) and micro-RNA (miRNA) [14, 15]. This has proven to be of significance in studies focused on understanding the pathogenesis of various malignancies [14, 16].

Although IHC and ISH have numerous advantages, the use of these techniques has been associated with certain limitations. The skills of the personnel involved in performing and interpreting IHC is a key factor governing the accuracy and reproducibility of this procedure [6, 8, 17]. The procedure can be associated with reaction bias when performing certain steps, including antigen retrieval and specimen fixation. Notably, antibodies may recognize similar epitopes on different protein targets, which can produce false positives. Interpretation bias is also possible when selecting an antibody panel and analyzing the results [18]. The use of FISH is also restricted by the availability of the probe, since the genetic aberration must be known hybridize the complimentary DNA/RNA sequence. For this reason, FISH cannot be utilized as a screening test for chromosomal aberrations [11, 19, 20]. Also notable, the size of a genomic aberration can also be a limiting factor [21]. Ultimately, utilizing ISH followed by IHC facilitates the complementary confirmation of RNA and protein signals, which provides assurance for questionable targets.

Several modifications to ISH and IHC have improved their accuracy over time [11, 22]. These modifications, combined with efforts such as the establishment of American Society of Clinical Oncology (ASCO) and College of American Pathologists (CAP) guidelines for HER2 testing in breast cancer and a standardized FDA approved IHC scoring system, have aided in increasing the clinical utility of these procedures [23].

The ability of IHC and FISH to identify specific molecular targets may potentially serve a role in identifying biomarkers with predictive, diagnostic and/or prognostic significance [24, 25]. This review will evaluate the role of IHC and FISH in predicting the response to treatment with targeted therapeutic agents. We will primarily focus on past studies that employed therapeutic agents against HER2, EGFR and PD-L1 in an attempt to analyze both successful and failed efforts that used IHC and/or FISH as predictive biomarkers for the success of these agents. Learning from the cases wherein predictive biomarkers have been successfully approved, we will explore the current scenario for biomarker development in FGFR and cMET targeted therapy. Our goal is to provide a better understanding of the use of IHC and FISH as predictive biomarkers, in order to improve the decision-making process concerning targeted therapy utilization for personalized cancer therapy.

## Need for robust predictive biomarkers

The concept of targeted cancer therapy aims at creating agents that agonize, antagonize or neutralize specific molecules on cancer cells. Certain tumors overexpress particular molecules that may be critical for survival, growth, proliferation or metastases of malignant tissues [26–28]. Various therapeutic agents such as tyrosine kinase inhibitors and growth inhibitory antibodies selectively target these molecules and eliminate sensitive cancer cell populations [29]. To maximize the clinical benefit of targeted therapy, it is crucial to accurately diagnose the presence of specific target molecules. IHC and FISH are considered as standard testing modalities for the evaluation of HER2 status in breast cancer cases, as per the ASCO/CAP 2013 recommendations [30]. Additionally, IHC and FISH have proven to be of value in determining the prognosis and predicting the response to therapy for agents targeting EGFR and PD-L1 [31–34]. The search for potential predictive biomarkers has been diversified to include anti-FGFR and anti-cMET targeted therapy agents.

## Trastuzumab/lapatinib in breast and gastric cancer

HER2 is overexpressed and/or amplified in several types of tumors including 10 – 30 % of gastric and gastroesophageal cancers, and 15 – 30% of breast cancer [35]. Other tumors that may possess increased HER2 levels include bladder-, head and neck-, ovarian-, endometrial-, colon-, lung- and uterine/cervical-cancers [27, 28, 36–40]. Similar findings from numerous other studies prompted the development of agents that specifically targeted HER2.

Trastuzumab is a monoclonal antibody specific to HER2 [41]. It inhibits PI3K/Akt and ras-Raf-MAPK signaling pathways by decreasing HER2 activity [42]. A review of data obtained from eight randomized controlled trials (RCTs) evaluating the use of trastuzumab in locally advanced breast cancer showed a hazard ratio (HR) for disease free survival (DFS) to be 0.60 (95% confidence interval (CI) 0.50 - 0.71, *P*<0.00001), and overall survival (OS) as 0.66 (95% CI 0.57 - 0.77, *P*<0.00001), strongly favoring treatment regimens utilizing trastuzumab [43]. A different trial, comparing the combination of doxorubicin, cyclophosphamide and docetaxel (AC-T) with combination docetaxel, carboplatin and trastuzumab (ACH), as well as AC-T plus trastuzumab in patients with HER2 positive early stage breast cancer, showed a considerable clinical benefit with the use of trastuzumab. The reported DFS rate at 5 years was 84% in the AC-T plus trastuzumab group, 81% in the TCH group and 75% in the AC-T only group. The OS rate was 92% in the AC-T plus trastuzumab group, 91% in the TCH group and 87% in the AC-T only group [44]. Another trial, called the joint analysis of National Surgical Adjuvant Breast and Bowel Project (NSABP B-31) and North Central Cancer treatment group (NCCTG N9831), compared combination doxorubicin, cyclophosphamide and paclitaxel with the same regimen plus trastuzumab. It showed a relative improvement in OS by 37% (HR of 0.63, 95% CI 0.54 - 0.73 and *P*<0.001) and an improvement in 10 year OS rate to 84% from 75.2% in the latter group. Additionally, the DFS improved by 40% (HR of 0.60, 95% CI 0.53 - 0.68 and *P*<0.001) and the 10 year DFS rate increased to 73.7% from 62.2% in the chemotherapy plus trastuzumab group [45]. Thus, it was evident that adjuvant trastuzumab therapy was associated with the improvement in OS and DFS. On the basis of the above and other similar evidence, the Food and Drug Administration (FDA) approved the use of trastuzumab as an adjuvant therapy agent for HER2 positive breast cancer in the year, 2006.

The key to achieving maximum therapeutic benefit with trastuzumab is an accurate diagnosis of HER2 overexpression and/or amplification. In view of this objective, ASCO/CAP issued guidelines for breast cancer in the year 2007 [46]. However, it was noted that there were certain shortcomings associated with recommended tests that may lead to misleading results. The limitations associated with IHC and FISH have been identified in various studies. Polysomy of chromosome 17 is responsible for an increased expression of HER2/neu protein in a minority of breast cancer cases with an absence of gene amplification [47]. IHC can falsely interpret chromosome 17 polysomy as an overexpression of HER2 [48]. It has also been found that tumors with low-grade HER2 gene amplification may have genetic heterogeneity that may influence the interpretation of HER2 status [49, 50]. HER2 genetic heterogeneity has been implicated in causing equivocal HER2 status when using FISH and has also been associated with HER2 IHC 1+/2+ [49, 51, 52]. Lastly, it has been observed that IHC and FISHfail to identify activating mutations of ERBB2 in the absence of HER2/ERBB2 gene amplification and/or overexpression [53].

The above findings prompted ASCO/CAP to bring forth a revised set of guidelines for evaluating HER2 status in patients with newly diagnosed invasive breast cancer. The new guidelines, issued in 2013, increased the HER2 positivity rate by 2% as compared to the previous year when the 2007 guidelines were in effect [23, 54] (Table 1). Thus, a greater number of patients may be able to receive targeted therapy with the implementation of the 2013 ASCO/CAP guidelines.

**Table 1:** Comparison of ASCO/CAP guidelines 2007 and update published in 2013 for HER2 positivity in breast cancer [45, 53]

Changes	ASCO/CAP 2007 guidelines	ASCO/CAP 2013 guidelines
Definition of HER2 positive status	IHC: **HER2 3+** (**> 30% invasive tumor cells** with intense and uniform staining); FISH: 1. Average number of HER2 copies **> 6 signals per nucleus** for tests with no internal control probe 2. HER2/CEP17 ratio **> 2.2**	IHC: **HER2 3+** (on basis of intense and complete circumferential membrane staining); ISH: 1. Dual probe HER2/CEP17 ratio **≥ 2.0** and average number of HER2 copies ≥ **4 signals per cell**. 2. Dual probe HER2/CEP17 ratio **≥ 2.0** and average number of HER2 copies **< 4 signals per cell**. 3. Dual probe HER2/CEP17 ratio **< 2.0** and average number of HER2 copies **≥ 6 signals per cell**. 4. Average **HER2 copies ≥ 6 signals per cell for single probe** system.
Definition of equivocal status for HER2	IHC: **HER2 2+**; FISH: 1. Average number of **HER2 copies from 4 to 6 signals per nucleus** for tests with no internal control probe 2. **HER2/CEP17 ratio: 1.8 to 2.2**	**Reflex test must be ordered** with alternative test on the same specimen, or either of the tests on a new specimen. IHC: 1. **HER2 2+** on basis of moderate/weak and/or incomplete circumferential membrane staining of > 10% invasive tumor cells. 2. **HER2 2+** on basis of intense, complete and circumferential staining of ≤ 10% invasive tumor cells; ISH: 1. **Single probe** system with average number of HER2 copies **< 6 and ≥ 4 signals per cell**. 2. **Dual probe** system with **HER2/CEP17 ratio of < 2.0** and average number of HER2 copies **< 6 and ≥ 4 signals per cell**.
Definition of HER2 negative status	IHC: 1. **HER2 0** (no staining) 2. **HER2 1+** (< 10% cells with weak and complete membrane staining) 3. **HER2 1+** (weak and incomplete membrane staining for any proportion of cells); FISH: 1. Average number of **HER2 copies < 4 signals per nucleus** for tests with no internal control probe 2. **HER2/CEP17 ratio < 1.8**	IHC: 1. **HER2 0** (≤ 10% invasive tumor cells with incomplete, faint membrane staining, or no staining) 2. **HER2 1+** (**> 10%** invasive tumor cells with faint and incomplete membrane staining); ISH: 1. **Single probe** system with average number of **HER2 copies < 4 signals per cell** 2. **Dual probe** system with **HER2/CEP17 ratio of < 2.0** and average number of HER2 copies < 4 signals per cell
Definition of indeterminate status for HER2	None defined	HER2 status should be reported as indeterminate if the test results cannot be classified into equivocal, positive or negative due to artifacts, analytic test failure or inadequate handling of specimen. A **new specimen should be obtained** and the cause of indeterminate result should be documented.
Criteria for interpretation of ISH	Using invasive tumor criteria, a minimum of 20 cells must be counted.	The ISH slide must be entirely scanned prior to counting 20 cells, or areas of HER2 amplification may be defined by using IHC. If visual estimation or image analysis of IHC or ISH slide reveals a second population of cells with increased HER2 signaling and >10% tumor cells, it warrants a separate count of at least 20 cells in this population and has to be reported. When using brightfield ISH, expert opinion must be obtained if the interpretation of the comparison of normal versus tumor cells is difficult due to artifactual patterns.
Criteria for interpretation of IHC	**More than 30%** of invasive tumor cells should have dark, circumferential and homogeneous pattern to report HER2 status as positive.	**More than 10%** of invasive tumor cells should have dark, circumferential and homogeneous pattern to report HER2 status as positive (3+ IHC).

Abbreviations: IHC: Immunohistochemistry; FISH: Fluorescent *in-situ* hybridization; ISH: *In-situ* hybridization; HER2: Human epidermal growth factor receptor 2; CEP17: Chromosome enumeration probe 17.

There are multiple points of distinction between the 2013 and 2007 ASCO/CAP guidelines for HER2 positivity in breast cancer. As per the 2013 guidelines, tumors with HER2 gene/ chromosome enumeration probe 17 (CEP17) ratio < 2.0 (≥ 2.2 in 2007 guidelines) together with average HER2 gene copies < 4.0 on dual probe ISH and/or IHC protein expression of 0 in ≤ 10% cells or 1+ in > 10% cells (> 30 % cells in 2007 guidelines) are diagnosed as HER2 negative. In cases of single probe ISH, HER2 negative status is defined by an average HER2 copy number of < 4.0. A positive HER2 status is diagnosed by IHC protein expression of 3+, defined as circumferential membrane staining that is intense and complete in > 10% cells. In case of a dual probe ISH, HER2 positivity is defined as average HER2 gene copies ≥ 4.0 and HER2/CEP17 ratio ≥ 2.0. The test is also positive if average HER2 copies are ≥ 6.0 with a HER2/CEP17 ratio of < 2.0, and, if < 4.0 average HER2 gene copies are reported together with a HER2/CEP17 ratio of ≥ 2.0. For a single probe ISH test, HER2 gene copies ≥ 6.0 signals per cell qualify HER2 status as positive.

Reflex testing must be performed using a different assay if the test results are reported as equivocal. An equivocal HER2 status based upon IHC test is defined by either 2+ HER2 protein expression with an intense or complete staining in ≤ 10% invasive tumor cells, or a 2+ HER2 protein expression with moderate/weak or incomplete staining. In dual probe ISH, a HER2/CEP17 ratio < 2.0 associated with average HER2 gene copies ≥ 4.0 and < 6.0 is considered equivocal. For single probe ISH, an equivocal result is defined by ≥ 4.0 and < 6.0 average HER2 copy number.

The new guidelines suggest three levels of testing for HER2-negative patients but exhibit histopathological discordance. In a patient with newly diagnosed breast cancer, a surgical specimen or a core biopsy can be used for performing HER2 testing. If discordance is present, the excisional specimen of the tumor should be tested. If the test is negative and there are persistent concerns regarding the reliability of the result, the test may be repeated using a different block of the tumor specimen. No further testing is required if all three tests are reported as negative.

The 2013 ASCO/CAP guidelines present several recommendations for optimizing the use of bright-field ISH in HER2 testing. It has been suggested that the specimens should be fixed within one hour of being retrieved. Additionally, the acceptable time-frame for specimen processing has been revised to between 6 – 72 hours (previously 6 – 48 hours).

AQUA, or automated quantitative analysis, is one of the latest technologies that have been conceived in an effort to meet ASCO/CAP guidelines. The system uses advanced image analysis algorithms and automated fluorescence microscopy for objectively providing continuous protein expression scores [55]. Consequently, the observer bias in traditional IHC is not present in AQUA analysis. Studies evaluating AQUA analysis in breast cancer have demonstrated that it can meet ASCO/CAP guidelines and thus potentially be used for standardized HER2 testing [56]. Further, data from one study reported that HER2 AQUA analysis (AQUA tissue microarray analysis) was more predictive of response to trastuzumab in metastatic breast cancer, as compared to whole slide FISH or IHC [57]. Nevertheless, large scale validation studies are warranted for adopting HER2 AQUA analysis for use in identifying a predictive biomarker for trastuzumab in breast cancer.

Recent evidence suggests that anti-HER2 agents may be of therapeutic value in patients diagnosed with advanced gastric cancer [58, 59]. Studies evaluating biopsy and surgical specimens of gastric tumors have demonstrated HER2 overexpression and gene amplification in a significant proportion of cases [60, 61].

A considerable amount of discrepancy was noted in HER2 status for gastric tumors when comparing results of IHC and FISH, performed per current guidelines. This was ascribed to incomplete membranous reactivity resulting from basolateral membranous immunoreactivity of glandular cells. Another possible reason may be tumor heterogeneity, found to be higher in gastric cancer when compared to breast cancer [62, 63]. A substantial inter-laboratory variation in scoring for equivocal HER2 IHC 2+ results has also been reported [63]. As a consequence of the inconsistency of IHC/FISH results, it was necessary to develop new guidelines for IHC/FISH scoring to accurately assess HER2 status in gastric tumors.

The trastuzumab for gastric cancer (ToGA) study is a phase III RCT involving patients diagnosed with gastric or gastroesophageal junction cancer. This trial involved the use of modified criteria to determine HER2 status, derived from the IHC scoring system for HER2 in breast cancer. As per the modifications, a strong and incomplete membranous staining was considered a 3+ score (positive), and the 10% area cut-off for membrane staining was to be disregarded [64]. Therefore, the inclusion criteria for study participants was positivity for HER2 overexpression as defined by IHC 3+ or FISH positive (HER2/CEP17 ratio of 2.0) and IHC 2+. The study involved randomization of the participants in a 1:1 ratio to two treatment groups; chemotherapy alone or chemotherapy plus trastuzumab. The median OS in the group receiving chemotherapy plus trastuzumab was 13.8 months (HR: 0.74; *P*=0.0046) versus 11.1 months for the treatment group that received chemotherapy alone. Thus, the use of an anti-HER2 agent in patients with HER2 positive advanced gastric cancer was found to decrease the risk of death by 26% [65]. Data showed that 15.7% of IHC 1+ and 4.9% IHC 0 cases were FISH positive, while 5% of IHC 3+ cases were FISH negative. The concordance rate of FISH and IHC was 87.2% with the use of the modified scoring system for HER2 status [64].

In addition to the primary goal, the ToGA study helped achieve several other significant objectives. The modified scoring system for HER2 status used in the study was made more reliable and reproducible by adding recommendations to standardize the procedure [66]. The study also showed that the modified scoring system for HER2 status in advanced gastric cancer was predictive of response to a trastuzumab-based treatment regimen [67, 68].

In recent years, lapatinib has emerged as a potent anti-cancer agent. Meta-analysis of three phase III RCTs evaluating the effects of lapatinib in HER2 positive versus negative metastatic breast cancer showed an improvement in progression free survival (PFS) and OS of patients with HER2 positive disease [69]. There is pre-clinical evidence demonstrating dual HER2/EGFR inhibition (NCI-N87 and SNU-216 cell lines) and apoptosis (NCI-N87 only) in gastric cancer cell lines with the use of lapatinib [70]. Lapatinib has exhibited synergistic anti-cancer action with multiple agents in pre-clinical models [70, 71]. Of note, it demonstrated synergistic cell growth inhibition with trastuzumab in HER2 amplified human gastric cancer cell lines [72]. Data obtained from a phase III RCT (EFG104900) showed significant improvement in OS and PFS with the use of combination lapatinib and trastuzumab versus lapatinib monotherapy in HER2 positive metastatic breast cancer patients [73]. Another phase III RCT, the TyTAN study, evaluated the clinical outcome of lapatinib plus paclitaxel versus paclitaxel monotherapy in patients diagnosed with HER2-amplified advanced gastric cancer. HER2 positivity was determined by using FISH for all study participants. All participants also underwent HER2 IHC testing for analysis purposes. The median OS and overall response rate (ORR) were higher with combination paclitaxel plus lapatinib versus paclitaxel monotherapy. Among the study participants receiving combination therapy, a difference in the response to treatment was directly correlated to the HER2 IHC score. Patients with IHC 3+ had a better response to combination therapy compared those with IHC 0/1+ and IHC 2+ [74]. Thus, in addition to demonstrating the activity of lapatinib plus paclitaxel, the TyTAN study also made crucial observations that have brought us closer to devising standardized guidelines for HER2 IHC status positivity in gastric cancer.

There is preliminary data suggesting that clinical outcomes with lapatinib may be influenced by population characteristics. A phase III RCT (TRIO-013/LOGiC-A trial) evaluated lapatinib in combination with capecitabine plus oxaliplatin (CapeOx) for HER2 positive advanced gastric, esophageal and gastroesophageal adenocarcinomas [75]. The study reported no significant benefit in median OS and PFS with lapatinib as compared to placebo. The HER2 IHC status exhibited no association with survival outcomes. However, a subgroup analysis exhibited higher OS in patients of younger age group and Asian ethnicity that received combination lapatinib plus CapeOx [75]. From these observations, it may be inferred that demographic factors such as age and ethnicity may have a bearing on clinical outcomes with lapatinib based therapy. Further research is needed to conclusively determine the effects of demographic influences on survival benefit with lapatinib.

## Cetuximab

EGFR, also known as HER1, is a member of the ERBB receptor tyrosine kinase family. Ligand binding induces homodimerization or heterodimerization of EGFR with other members of the ERBB family and leads to downstream signaling through the mitogen activated protein (MAP) kinase and PI3K/Akt pathways [76]. Various studies have implicated the overexpression of EGFR in the progression of cancer [77, 78]. The majority of tumors associated with overexpression of EGFR originate from pancreatic, colorectal, breast and lung tissues [79–83]. EGFR-targeting agents may prove to be of value in such malignancies.

Cetuximab is an anti-EGFR chimeric monoclonal antibody. It binds to the extracellular ligand binding domain of EGFR, consequently blocking downstream signaling pathways associated with EGFR [84, 85]. It also augments internalization and later degradation of the EGFR receptor [85]. In addition, cetuximab has exhibited complement-mediated cytotoxicity and antibody-dependent cellular cytotoxicity (ADCC) [86, 87]. These properties have shown clinical utility in the treatment of several malignancies.

Cetuximab has been approved by the FDA for use in several malignancies. In 2004, cetuximab was first approved for use in combination with irinotecan for EGFR positive metastatic colorectal cancer (mCRC) refractory to irinotecan based therapy [88] (Table 2). Single agent use of cetuximab in EGFR positive mCRC is approved for patients that failed both oxaliplatin and irinotecan based therapy, and for those presenting with recurrent disease along with intolerance to irinotecan based therapy [88–91]. In K-ras wild type, EGFR positive mCRC, combination cetuximab plus FOLFIRI (irinotecan, leucovorin and 5-fluorouracil) is approved as first line therapy [92].

**Table 2:** List of clinical trials that assessed EGFR IHC and/or EGFR FISH as biomarkers when evaluating targeted therapy agents for EGFR positive malignancies

Drug	Treatment regimen	Biomarker	Tumor histology	Criteria for biomarker positivity	Percent of tumor samples expressing the biomarker	Outcome	Reference
Cetuximab	Cetuximab 400 mg/m^2^ D1, then 250 mg/m^2^ weekly; cisplatin 80 mg/m^2^, vinorelbine 25 mg/m^2^	EGFR IHC	NSCLC	EGFR IHC score ≥200 categorized as high and <200 as low, on a scale of 0-300	High: 31% (345 of 1121 patients) Low: 69% (776/221 patients)	High EGFR expression group: - Cetuximab plus chemotherapy: Median OS of 12 months (95% CI: 10.2 - 15.2) - Chemotherapy alone: Median OS of 9.6 months (95% CI: 7.6 – 10.6) Low EGFR expression group: - Cetuximab plus chemotherapy: Median OS of 9.8 months (95% CI: 8.9 – 12.2) - Chemotherapy alone: Median OS of 10.3 months (95% CI: 9.2 – 11.5) Evaluation of the difference in HR for high (HR 0·73, 0·58-0·93; p=0·011) and low EGFR expression group (HR 0·99, 0·84-1·16; p=0·88) through a treatment interaction test exhibited predictive value for EGFR expression (p= 0.044)	FLEX study [231]
Cetuximab	Cetuximab with or without T/C	EGFR FISH, EGFR IHC	NSCLC	EGFR FISH positivity by Colorado scoring system; EGFR IHC positivity: ≥1 tumor cell exhibiting staining	EGFR FISH: 51.9% (54 of 104 patients) EGFR IHC: 88.5% (131 of 148 patients)	EGFR FISH for cetuximab plus T/C cohort: -No significant difference noted in PFS of FISH positive versus FISH negative cases (HR: 0.99, p= 0.97) -OS was shorter in FISH positive versus FISH negative patients (HR: 1.07, p= 0.81) -ORR was 37% in FISH positive, as compared to 30.8& in FISH negative patients EGFR IHC for cetuximab plus T/C cohort: -PFS was significantly lower for IHC positive versus IHC negative cases (HR: 1.81, p= 0.15) - No difference noted in OS for IHC positive versus negative cases (HR: 1.00, p= 0.99) - The difference between ORR of IHC positive and negative cases was non-significant (p= 0.49)	BMS099 study [127]
Cetuximab	Cetuximab 400 mg/m^2^ D1, then 250 mg/m^2^ weekly; carboplatin (area under curve = 6), paclitaxel 225 mg/m^2^	EGFR FISH	NSCLC	EGFR FISH showing ≥4 gene copies in each cell in ≥40% cells, or, gene amplification as defined by ≥15 copies of the gene in ≥10% cells, presence of a gene cluster or a gene-chromosome ratio ≥2	59.2% (45 of 76 patients)	- Median survival time for FISH positive cases was 15 months, versus 7 months for FISH negative cases (p= 0.04) - Median PFS for FISH positive cases was 6 months, versus 3 months for FISH negative cases (p= 0.0008). - Disease control rate was 81% in FISH positive versus 55% in FISH negative cases (p= 0.02). - Response rate of 45% was observed in FISH positive cases, versus 26% in FISH negative cases (p= 0.14)	[232]
Cetuximab	Single agent cetuximab 400 mg/m^2^ D1, then 250 mg/m^2^ weekly; Platinum plus cetuximab salvage therapy if disease progression on cetuximab monotherapy	EGFR IHC	SCCHN	EGFR IHC score 1+, 2+ or 3+ on a scale of 0 to 3+, using a standardized IHC assay	97% (97 of 100 patients)	Single agent phase: - RR: 13% - Disease control rate: 46% - Median time to progression: 70 days Cetuximab plus platinum based therapy phase: - Objective RR: zero - Disease control rate: 26% - Median time to progression: 50 days	[92]
Cetuximab	Cetuximab 400 mg/m^2^ D1, then 250 mg/m^2^ weekly; fluorouracil 1000 mg/m^2^, cisplatin 100 mg/m^2^ or carboplatin (area under curve = 5)	EGFR IHC	SCCHN	EGFR IHC results stratified in 3 groups according to percentage of cells testing positive for EGFR: 0%, >0 to <40% and ≥40%; no criteria for positivity defined	98% (405 of 413 patients) had detectable EGFR; - 1.9% (8 of 413 patients) with 0% cells testing positive for EGFR; - 15.4% (64 of 413 patients) with >0 to <40% cells testing positive for EGFR; - 82.5% (341 of 413 patients) with ≥40% cells testing positive for EGFR	>0 to<40% cells positive for EGFR: - Median OS 10.9 months with cetuximab plus chemotherapy versus 7.8 months for chemotherapy alone (HR: 0.72, 95% CI= 0.4 to 1.28) - Median PFS 5.7 months for cetuximab plus chemotherapy versus 4.1 months for chemotherapy alone (HR: 0.6, 95% CI= 0.33 to 1.08) ≥40% cells positive for EGFR: - Median OS 10.1 months for cetuximab plus chemotherapy versus 7.1 months for chemotherapy alone (HR: 0.75, 95% CI= 0.59 to 0.95) - Median PFS 5.7 months for cetuximab plus chemotherapy versus 3.1 months for chemotherapy alone (HR: 0.47, 95% CI= 0.37 to 0.61)	[94]
Cetuximab	Cetuximab 400 mg/m^2^ D1, then 250 mg/m^2^ weekly with or without high-dose radiotherapy	EGFR IHC	SCCHN	EGFR IHC results stratified in groups according to percentage of cells testing positive for EGFR: ≤50% and >50%, unknown and undetectable; no criteria for positivity defined	99% (421 of 424 patients) had detectable EGFR staining; - 42.4% (180 of 424 patients) with ≤50% cells testing positive for EGFR - 36.8% (156 of 424 patients) with >50% cells testing positive for EGFR - 20% (85 of 424 patients) with unknown percentage of cells testing positive for EGFR - <1% (3 of 424 patients) with undetectable EGFR	PFS: - Cetuximab plus radiotherapy: 17.1 months - Radiotherapy alone: 12.4 months (HR: 0.70, 95%CI: 0.54 to 0.90, p= 0.006) OS: - Cetuximab plus radiotherapy: 49 months - Radiotherapy alone: 29.3 months (HR: 0.74, 95%CI: 0.57 to 0.97, p= 0.03)	[93]
Cetuximab	Single agent cetuximab 400 mg/m^2^ D1, then 250 mg/m^2^ weekly	EGFR IHC	mCRC	Definition for a **negative** EGFR IHC (as per study design): Absent membrane staining in ≥500 tumor cells	- 8.2% (7 of 85 patients) tested **negative** for EGFR IHC - 91.8% (78 of 85 patients) tested positive for EGFR IHC	- Patients **negative** for EGFR eligible for participation as per study design - Median TTP: 2.5 months - Median OS: 10 months	[98]
Cetuximab	cetuximab 400 mg/m^2^ D1, then 250 mg/m^2^ weekly; irinotecan 180 mg/m^2^ every 2 weeks or 125 mg/m^2^ weekly plus fluorouracil and leucovorin; single agent irinotecan as 350 mg/m^2^ every three weeks	EGFR IHC	mCRC	EGFR IHC scoring using a standard IHC assay; categorized in 4 groups on basis of percentage of cells positive for EGFR: 0 to <10%, 10 to <20%, 20 to 40% and ≥40%	- 41% (135 of 329 patients) with 0 to <10% cells positive for EGFR - 13.7% (45 of 329) with 10 to <20% cells positive for EGFR - 16.7% (55 of 329) with 20 to 40% cells positive for EGFR - 28.6% (94 of 329) ≥40% cells positive for EGFR	Rate of response with cetuximab plus irinotecan: - 22.9% (25 of 109 patients) with ≤10% cells positive for EGFR - 20% (4 of 20) with >10 to ≤20% cells positive for EGFR - 22.2% (6 of 27) with >20 to ≤35% cells positive for EGFR - 24.2% (15 of 62) with ≥35% cells positive for EGFR Rate of response with cetuximab monotherapy: - 7.1% (4 of 56 patients) with ≤10% cells positive for EGFR - 31.3% (5 of 16) with >10 to ≤20% cells positive for EGFR - 0% (0 of 7) with >20 to ≤35% cells positive for EGFR - 9.4% (3 of 32) with ≥35% cells positive for EGFR	[233]
Cetuximab	Cetuximab 400 mg/m^2^ D1, then 250 mg/m^2^ weekly	EGFR IHC	mCRC	EGFR IHC scoring using a standard IHC assay: -Undetectable -1+: faint membrane staining -2+: weak to moderate staining -3+: strong and complete staining -Unknown	- 2.6% (9 of 346 patients) with undetectable EGFR expression - 59.8% (207 of 346 patients) with 1+ EGFR IHC score - 28.3% (98 of 346) with 2+ EGFR IHC score - 9% (31 of 346) with 3+ EGFR IHC score - 0.3% (1 of 346 patients) with unknown EGFR IHC expression	Outcome (partial response, PR) stratified as per EGFR IHC score: - undetectable: 11.1% (1 of 9 patients with PR, 95%CI: 0.3 to 48.2) - 1+: 12.1% (25 of 207 patients with PR, 95%CI: 8 to 17.3) - 2+: 8.2% (8 of 98 patients with PR, 95%CI: 3.6 to 15.5) - 3+: 19.4% (6 of 31 patients with PR, 95%CI: 7.5 to 37.5)	[88]
Cetuximab	Cetuximab 400 mg/m^2^ D1, then 250 mg/m^2^ weekly	EGFR IHC	CRC	EGFR IHC scoring using a standard IHC assay: 0, 1+, 2+ and 3+. A positive EGFR IHC result was defined as a score >0.	All candidates required to have EGFR IHC score >0 for enrollment.	Outcome (treatment responders) stratified as per EGFR IHC score: - 0: not eligible for participation in study - 1+: 6% (1 of 17 patients) - 2+: 13% (4 of 30 patients) - 3+: 0% (0 of 10 patients) Partial response observed in 9% (5 of 57 patients, 95%CI: 0.03 to 0.19) Stable disease, for a minimum of 12 weeks, was observed in 37% (21 of 57 patients) cases	[89]
Cetuximab	Cetuximab 400 mg/m^2^ D1, then 250 mg/m^2^ weekly; irinotecan 350 mg/m^2^ at 3 weeks interval	EGFR IHC	mCRC	EGFR IHC scoring criteria: - None - 1+: weak staining - 2+: moderate staining - 3+: strong staining - Missing	All candidates required to have EGFR expression for eligibility.	Cetuximab plus irinotecan: - Median survival: 10.7 months (95%CI: 9.6 to 11.3) - Median PFS: 4 months (95%CI: 3.2 to 4.1) - ORR: 16.4% (95%CI: 13.6 to 19.4) Irinotecan alone: - Median survival: 10 months (95%CI: 9.1 to 11.3) - Median PFS: 2.6 months (95%CI: 2.1 to 2.7) - ORR: 4.2% (95%CI: 2.8 to 6.0)	EPIC trial [90]
Necitumumab	Necitumumab 800 mg D1 and D8, continued after completion of chemotherapy; cisplatin 75 mg/m^2^ D1; gemcitabine 1250 mg/m^2^ D1 and D8	EGFR FISH	Squamous NSCLC	EGFR FISH results determined using Colorado scoring system	51% of study participants evaluable for EGFR FISH status in exploratory analysis; 37.3% (208 patients) of the evaluated samples positive for EGFR FISH	Median OS: - Necitumumab plus chemotherapy: 11.5 months (95%CI: 10.4 to 12.6) - Chemotherapy alone: 9.9 months (95% CI: 8.9 to 11.1) EGFR FISH positive patients: - HR for OS: 0.70 - HR for PFS: 0.71 EGFR FISH negative patients: - HR for OS: 1.02 - HR for PFS: 1.04	[130, 133] SQUIRE trial

Abbreviations: EGFR, epithelial growth factor receptor; IHC, immunohistochemistry; FISH, fluorescent *in-situ* hybridization; NSCLC, non-small cell lung cancer; SCCHN, squamous cell carcinoma of head and neck; CRC, colorectal cancer; mCRC, metastatic colorectal cancer; HR, hazard ratio; CI, confidence interval; OS, overall survival; PFS, progression free survival; TTP, time to progression; RR, response rate; ORR, overall response rate; PR, partial response; T/C. taxanes/carboplatin; D1, day 1; D8, day 8.

In patients diagnosed with squamous cell carcinoma of the head and neck (SCCHN), cetuximab may be used as a single agent in cases with recurrent or metastatic disease that failed platinum based therapy [93] (Table 2). In cases of regionally or locally advanced SCCHN, cetuximab is approved for use in combination with radiotherapy [94]. For metastatic and/or recurrent locoregional SCCHN, cetuximab can be used in combination with 5-fluorouracil plus platinum based chemotherapy [95, 96].

To study the effects of cetuximab, several efforts were made to optimize case selection during the initial clinical studies. Based upon the Herceptin/HercepTest drug-diagnostic test association for trastuzumab (anti-HER2 agent), EGFR expression as determined by an IHC test was required for participants enrolled in clinical trials evaluating cetuximab based therapy [97]. However, review of data showed that patients with colorectal cancer diagnosed as EGFR-negative by IHC testing also responded to treatment with cetuximab [98]. Similar findings were demonstrated in a phase II trial evaluating the effects of cetuximab in refractory mCRC diagnosed as negative for EGFR by IHC testing. 8.2% (7 patients) of all study candidates (85 patients) showed major response with cetuximab monotherapy [99]. Thus, the predictive value of EGFR IHC testing for cetuximab based therapy in colorectal cancer was found to be questionable. In view of the above, the National Comprehensive Cancer Network (NCCN) issued guidelines stating that case selection for cetuximab therapy should not depend upon the results of EGFR IHC testing [100]. However, later studies suggested that using FISH to determine EGFR status may potentially be used as a predictive biomarker for cetuximab based therapy [101, 102].

Several theories have been investigated in the search for a predictive biomarker for cetuximab therapy. Data from a study that evaluated genetic changes in EGFR after treatment with cetuximab or panitumumab in mCRC cases put forth an interesting hypothesis. The dosage of cetuximab that led to inhibition of proliferation in mCRC cell populations with amplified numbers of EGFR copies showed no effect on cell populations lacking EGFR amplification. Thus, it was suggested that case-selection for anti-EGFR therapy may be based upon the number of EGFR gene copies [102]. However, another study involving mCRC patients that received cetuximab based therapy noted a response to treatment among cases that tested negative for EGFR gene amplification on FISH analysis. Therefore, this study suggested that the use of EGFR FISH analysis for selection of mCRC cases that would receive cetuximab was found to be of little value [103].

The KRAS gene is of particular interest in cetuximab based treatment regimens. It belongs to the RAS gene family that produces GDP/GTP binding proteins. RAS plays a crucial role in normal downstream cellular signaling of cell surface receptors for functions related to growth and senescence. Several RAS gene mutations have been associated with oncogenesis [104, 105]. Typically, RAS mutations occur at codons 12, 13 or 61 [106]. KRAS, is specifically mutated at codons 12 or 13 of exon 2 [107–109]. Both KRAS mutations lead to impairment of GTPase activating protein (GAP) binding, leading to constitutive activation [106]. Almost 35-40% of mCRC patients have been reported to have activating mutations in KRAS. These mutations have been associated with activation of intracellular signal transduction pathways through EGFR-independent mechanisms [109].

There is a significant amount of evidence suggesting that KRAS mutation status may be predictive of resistance to cetuximab based therapy in mCRC patients. Several clinical trials and single arm studies involving patients diagnosed with mCRC have demonstrated a direct association between KRAS mutations and resistance to treatment with cetuximab [110–118]. Further studies evaluating specific KRAS mutations and their association with OS and PFS upon treatment with cetuximab have made further observations. Chemotherapy-refractory mCRC cases positive for KRAS mutation at codon 13 showed less resistance to cetuximab therapy [119]. Thus, it appears that specific KRAS mutations may have greater predictive value over others when we attempt to determine response to cetuximab based therapy. In view of the above evidence, the American Society of Clinical Oncology (ASCO) put forth guidelines in 2009 stating that all mCRC patients must undergo tests for KRAS mutations in their tumor tissue prior to initiating treatment with anti-EGFR antibodies. A positive signal for KRAS mutation at codon 12 or 13 shall exclude treatment with any anti-EGFR monoclonal antibody [120].

Extensive research on members of the RAS family has implicated several other crucial mutations that are predictive for resistance to treatment with anti-EGFR antibodies. The CRYSTAL study showed an improvement in objective response, OS and PFS after addition of cetuximab to FOLFIRI versus FOLFIRI alone, in KRAS wild type mCRC patients [121]. DNA samples collected during the study determined KRAS exon 2 mutation status and were re-analyzed for other RAS mutations. These included NRAS codons at exons 2, 3 and 4, and KRAS codons at exons 3 and 4. It was observed that patients with extended RAS mutations showed no benefit in OS, PFS or objective response upon addition of cetuximab to FOLFIRI versus FOLFIRI alone [122]. Similarly, retrospective analysis of the PRIME study demonstrated a lack of response in mCRC patients with extended RAS mutations upon treatment with the combination of panitumumab plus FOLFOX4 (leucovorin, oxaliplatin and fluorouracil) [123]. ASCO guidelines were therefore recently revised to include mutation testing for both NRAS and KRAS at exon 4 (codons 146 and 117), exon 3 (codons 61 and 59) and exon 2 (codons 13 and 12) for all mCRC cases that are being considered for treatment with anti-EGFR monoclonal antibodies [124].

Cetuximab has been evaluated as a potential treatment option for advanced non-small cell lung cancer (NSCLC) (Table 2). The first line Erbitux in lung cancer (FLEX) study was a phase III clinical trial assessing the effects of cetuximab plus first-line chemotherapy (cisplatin and vinorelbine) in advanced EGFR positive NSCLC cases. The combination therapy was associated with a significant improvement in OS, as compared to cisplatin plus vinorelbine alone [123]. Another phase III RCT, BMS099, reported significant improvement in ORR with cetuximab plus chemotherapy (carboplatin plus paclitaxel/docetaxel) versus chemotherapy alone in advanced NSCLC cases [125]. Data gathered during FLEX and BMC099 phase III clinical trials prompted further investigation to determine suitable predictive biomarkers for improvement in outcomes of advanced NSCLC patients.

Numerous endeavors have been made to identify a predictive biomarker for the cetuximab plus chemotherapy treatment regimen in advanced NSCLC. Treatment outcomes of participants involved in the FLEX trial were retrospectively analyzed in association with the status of several biomarkers [126]. Formalin fixed paraffin embedded (FFPE) tissue samples from the study participants were analyzed for KRAS codons 12 and 13 mutations by PCR assay and PTEN expression through IHC and EGFR copy number by dual color FISH. However, it was found that all biomarkers evaluated in this study had no predictive value for treatment efficacy of combination cetuximab plus chemotherapy [126]. Another study assessed the tumor EGFR IHC data collected during the FLEX study and compared cases with high (IHC score ≥ 200 on a scale of 0-300) versus low (IHC score < 200 on a scale of 0-300) tumor EGFR expression [127]. It was observed that patients with high EGFR expression exhibited a significant improvement in OS when treated with combination cetuximab plus chemotherapy versus chemotherapy alone. Conversely, a shorter median OS was noted with cetuximab plus chemotherapy treatment as compared to chemotherapy alone in patients with low EGFR expression. Thus, a high tumor EGFR expression may potentially be predictive of survival benefit from cetuximab based therapy for advanced NSCLC cases [127]. The survival indices in high and low EGFR expression groups were further analyzed to search for possible association with tumor EGFR mutation status. However, it was demonstrated that EGFR mutation status did not limit the improvement in OS for advanced NSCLC with high EGFR expression upon treatment with combination cetuximab plus chemotherapy (oxaliplatin plus vinorelbine) [123]. Much the same as FLEX study based trials, tumor samples obtained from BMS099 clinical trial participants were also evaluated for several predictive biomarkers. This consisted of FISH analysis of EGFR gene copy number, IHC for EGFR protein expression and direct sequencing for EGFR and KRAS mutations. None of the biomarkers assessed in the study exhibited association with ORR, OS or PFS [128].

Contiguous with the studies based on data from FLEX and BMS099 clinical trials, a phase II selection trial involving advanced NSCLC cases receiving cetuximab plus chemotherapy (carboplatin plus paclitaxel) assessed patients for EGFR status using FISH in association with survival outcomes [101]. The criteria for EGFR FISH positivity was established as ≥ 4 gene copies per cell in ≥ 40% cells. Alternatively, EGFR FISH could also be classified as positive based on gene amplification, defined by ≥ 15 copies of the gene in ≥ 10% cells, presence of a gene cluster or a gene-chromosome ratio ≥ 2. The study reported a significant survival benefit (median survival time and PFS) in the FISH positive group as compared to the FISH negative group. Additionally, the FISH positive group had statistically superior disease control rate and a higher number of treatment responders (partial and complete response) as compared to the FISH negative group [101]. These observations have collectively presented evidence suggesting that EGFR FISH may be used as a predictive biomarker for cetuximab based therapy in advanced NSCLC. However, large scale clinical studies are warranted in order to validate the above findings.

The SWOG S0819 was a phase III clinical trial with a primary focus on the evaluation of cetuximab efficacy in combination with chemotherapy for the treatment of advanced NSCLC. Additionally, this trial also assessed EGFR FISH as a predictive biomarker for cetuximab based treatment regimens in advanced NSCLC patients [33]. Positive results for EGFR gene amplification by FISH were defined using the Colorado scoring system. According to this system, specimens with ≥ 4 copies of EGFR signal in ≥ 40% cells, or ≥ 4 spots of gene clusters or ≥ 10% of tumor cells with ≥ 15 copies of EGFR signals or an EGFR/CEP7 ratio ≥ 2 were classified as positive for EGFR [129]. Although no overall improvement in OS was reported for the entire study population, a subgroup analysis demonstrated OS benefit in FISH positive squamous cell NSCLC patients (n= 321, HR= 0.56, *P*= 0.006) [129]. Therefore, it can be stated that these findings are indicative of the predictive significance of EGFR FISH in squamous cell NSCLC.

With a background of aforementioned studies that provided an assessment of the role of cetuximab in therapy for advanced NSCLC and potential predictive biomarkers, it has become imperative to attempt further validation of this data with biomarker driven prospective studies.

## Necitumumab

Necitumumab is another recombinant human monoclonal EGFR antibody [130, 131]. The efficacy of the drug in non-squamous cell and squamous cell NSCLC have been evaluated in the INSPIRE and SQUIRE clinical trials respectively [131–133]. The SQUIRE trial also evaluated potential predictive biomarkers for necitumumab.

The INSPIRE trial was a phase III RCT that assessed the efficacy of necitumumab plus pemetrexed-cisplatin combination versus pemetrexed-cisplatin combination alone in patients with stage IV non-squamous cell NSCLC [132]. This study concluded that necitumumab plus pemetrexed-cisplatin combination had no survival benefit over pemetrexed-cisplatin therapy alone [132].

The SQUIRE trial was a phase III RCT that evaluated necitumumab plus gemcitabine-cisplatin combination versus gemcitabine-cisplatin alone in stage IV squamous NSCLC [131]. A significant benefit in OS was observed with necitumumab plus gemcitabine-cisplatin combination (median OS: 11.5 months; 95% CI: 10.4 – 12.6) as compared to the control group (median OS: 9.9 months; 95% CI: 8.9 – 11.1) [131]. In addition to the above, recently published results from an exploratory analysis of archived tumor samples from the SQUIRE trial have reported outcomes favoring the use of EGFR FISH in squamous NSCLC [134]. Tumor samples were available from 51% of study participants, of which 37.3% (208 patients) were EGFR FISH positive. The treatment HR for OS and PFS in FISH positive patients was recorded as 0.70 and 0.71 versus 1.02 and 1.04 for FISH negative patients, respectively [134]. Thus, it may be suggested that EGFR gene copy number analysis by FISH can potentially be employed as a predictive biomarker for necitumumab in squamous cell NSCLC. Further biomarker-driven clinical trials are necessary to conclusively validate these findings.

On the basis of the observations made in the SQUIRE trial, the US FDA approved necitumumab plus gemcitabine-cisplatin combination for use as a first-line therapy option in metastatic squamous cell NSCLC [135]. Additionally, necitumumab has also been authorized for use by the European Commission (the Committee for Medicinal Products for Human Use; CHMP) for advanced squamous NSCLC in combination with gemcitabine plus cisplatin [136]. Of note, in contrast to the US FDA approval, European Commission limited the approved indication to advanced squamous NSCLC with EGFR expression although they did not specify the methods for testing the expression of EGFR [136]. This is a good example where a clinically meaningful biomarker based subgroup analysis influenced actual approved indication of the treatment regimen.

## cMET receptor protein

cMET is a membrane spanning receptor tyrosine kinase that binds to hepatocyte growth factor (HGF) [137]. The binding of HGF/MET leads to activation of multiple downstream molecules through PI3K/AKT, NFκB, MAPK and STAT signaling pathways [138–141]. MET mediates several cellular processes that are stimulated by hepatocyte growth factor/scatter factor (HGF/SF) such as proliferation, motility and morphogenesis [142–144]. Upregulation of HGF/SF and MET genes after injury to organs such as the heart, liver and kidneys suggests that they may also have a role in tissue regeneration and homeostasis [145–148]. MET is gaining significance as a potential target in oncology as it plays a crucial role in mediation of numerous cellular processes [149].

Studies investigating pathogenesis of multiple malignancies have suggested that aberrations in MET may act as oncogenic drivers. These may include MET gene amplification, protein overexpression and MET gene mutation [150, 151]. Tumors of the stomach, breast, thyroid and lung are among several others that have been reported to exhibit MET aberrations [150–155]. MET has also been implicated in resistance to VEGFR and EGFR inhibitor based therapy [149]. Considering the role of the MET pathway in oncogenesis, there is an increasing emphasis on exploring prospects of MET- targeting therapy in several malignancies, together with identification of potential biomarkers for the same.

Efforts undertaken to develop predictive biomarkers and evaluate the efficacy of MET targeting agents have accrued initial success (Table 3). Numerous MET targeting agents, mostly either small molecules or monoclonal antibodies have been developed and are presently being evaluated for their efficacy in several malignancies [149]. A phase II RCT evaluating combination onartuzumab (MET antagonist) plus erlotinib in advanced NSCLC used IHC for diagnosing MET status [156]. MET IHC positive patients receiving onartuzumab plus erlotinib exhibited improvement in OS and PFS. On the other hand, MET IHC negative patients receiving the same combination showed worse survival outcomes when compared to the MET IHC positive group [156]. On account of these findings, it is prudent to further investigate MET IHC as a predictive biomarker for MET antagonist therapy in advanced NSCLC patients.

**Table 3:** List of clinical trials that assessed MET IHC as a biomarker when evaluating targeted therapy agents for MET positive malignancies

Drug	Treatment regimen	Biomarker	Tumor histology	Criteria for biomarker positivity	Percent of tumor samples expressing the biomarker	Outcome	Reference
Onartuzumab	Onartuzumab 15 mg/kg every 3 weeks; erlotinib 150 mg/day	MET IHC	NSCLC	MET IHC scoring: - 0: less than 50% cells staining with any intensity, or no staining - 1+: <50% cells with moderate/high and ≥50% cells with weak/high intensity staining - 2+: <50% with strong and ≥50% cells with moderate/high intensity staining - 3+: ≥50% cells with strong intensity staining - MET positivity defined as a MET IHC score of 2+ or 3+	- 52% (66 of 128 patients)	Risk of disease progression: - MET positive sub-group: 47% decrease associated with the use of onartuzumab plus erlotinib, median 2.9 months versus 1.5 months with placebo plus erlotinib (HR: 0.53, p= 0.04) - MET negative sub-group: early progression associated with the use of onartuzumab plus erlotinib, median 1.4 months versus 2.7 months with placebo plus erlotinib (HR: 1.82, p= 0.05) Median OS sub-group analysis: - MET positive sub-group: Increased survival associated with the use of onartuzumab plus erlotinib, median 12.6 months versus 3.8 months with placebo plus erlotinib (HR: 0.37, p= 0.002) - MET negative sub-group: Lower survival benefit associated with the use of onartuzumab plus erlotinib, median 8.1 months versus 15.3 months with placebo plus erlotinib (HR: 1.78, p= 0.16) MET IHC evaluation: - Benefit in OS (HR: 0.52, p= 0.023) and PFS (HR: 0.78, p= 0.317) was found to decrease with a cut-off value of ≥10% (percent cells with moderate to strong intensity staining), as compared to cut-off value of ≥50%. - A cut-off value of ≥90% was associated with benefit in OS (HR: 0.3, p= 0.001) and PFS (HR: 0.47, p= 0.028) that was comparable to the results observed with a cut-off value of ≥50%. - Benefit in PFS and OS was maintained with the use of onartuzumab in patients with MET IHC score of 2+ and 3+. - The use of onartuzumab in patients with MET IHC score of 0 and 1+ was associated with poorer outcomes in OS and PFS.	[155]
Onartuzumab	Onartuzumab with/without mFOLFOX6	MET IHC	Gastric/ GEJ cancer	MET IHC score 1+, 2+ or 3+ on a scale of 0 to 3+, using a standard IHC assay	All candidates required to be positive for MET expression by IHC for enrollment in study.	ITT group median OS: - Onartuzumab plus mFOLFOX6: 11 months - Placebo plus mFOLFOX6: 11.3 months (HR: 0.82, p= 0.244) ITT group median PFS: - Onartuzumab plus mFOLFOX6: 6.7 months - Placebo plus mFOLFOX6: 6.8 months (HR: 0.90, p= 0.429) MET IHC 2+/3+ group median OS: - Onartuzumab plus mFOLFOX6: 11 months - Placebo plus mFOLFOX6: 9.7 months (HR: 0.64, p= 0.062) MET IHC 2+/3+ group median PFS: - Onartuzumab plus mFOLFOX6: 6.9 months - Placebo plus mFOLFOX6: 5.7 months (HR: 0.79, p= 0.223)	[234]
Rilotumumab	Rilotumumab 15 mg/kg D1 every 3 weeks; epirubicin 50 mg/m^2^ D1, capecitabine 625 mg/m^2^ BID D1-21, cisplatin 60 mg/m^2^ D1	MET IHC	Gastric/ GEJ cancer	MET IHC score 1+, 2+ or 3+ on a scale of 0 to 3+, using a standard IHC assay	All candidates required to be positive for MET expression by IHC for enrollment in study.	Early termination of study due to greater mortality in rilotumumab plus ECX group (128 deaths) versus placebo plus ECX (107 deaths) Median PFS: - Rilotumumab plus ECX: 5.7 months - Placebo plus ECX: 5.7 months Median OS: - Rilotumumab plus ECX: 9.6 months - Placebo plus ECX: 11.5 months	RILOMET 1 study [158]
Rilotumumab	Rilotumumab 15 mg/kg D1 every 3 weeks; capecitabine 1000 mg/m^2^ BID D1-14, cisplatin 80 mg/m^2^ D1	MET IHC	Gastric/ GEJ cancer	MET IHC score 1+, 2+ or 3+ on a scale of 0 to 3+, using a standard IHC assay	All candidates required to be positive for MET expression by IHC for enrollment in study.	Early termination of study due to greater mortality with rilotumumab plus CX versus placebo plus CX [235]	RILOMET 2 study [159]

Abbreviations: MET, Hepatocyte growth factor receptor; IHC, immunohistochemistry; NSCLC, non-small cell lung cancer; GEJ, Gastro-esophageal junction; ECX, epirubicin, cisplatin and capecitabine; CX, cisplatin plus capecitabine; ITT, intent to treat; HR, hazard ratio; CI, confidence interval; OS, overall survival; PFS, progression free survival; D1, day 1; BID, twice daily; mFOLFOX6, leucovorin, fluorouracil and oxaliplatin.

A limited number of clinical trials have been conducted to evaluate the role of MET pathway inhibitors and identify potential biomarkers in the treatment of advanced gastric and esophagogastric junction (EGJ) cancers. Rilotumumab is a monoclonal antibody that functions as an HGF/SF inhibitor, thus preventing downstream signaling through cMET pathway [157]. Data from a phase II RCT comparing rilotumumab plus chemotherapy (epirubicin, cisplatin and capecitabine; ECX) versus chemotherapy alone in locally advanced or metastatic gastric/EGJ cancer, was retrospectively analyzed to determine the association between MET pathway biomarkers and clinical outcomes [158]. The tumor samples collected during the trial were examined for MET protein expression by IHC and MET gene copy number by FISH. It was observed that MET gene copy number by FISH analysis showed no correlation with survival outcomes. However, patients with a high MET expression on IHC exhibited greater improvement in survival indices (OS and PFS) with rilotumumab plus chemotherapy treatment versus those with a low MET expression [158]. These findings served as a basis for further studies investigating the efficacy of rilotumumab in advanced gastric and esophagogastric cancers. In addition, data from this study provided initial evidence that suggested a potential use of MET IHC as a predictive biomarker in rilotumumab therapy.

Two phase III trials, RILOMET 1 and RILOMET 2, were initiated to evaluate the efficacy of rilotumumab in combination with cisplatin and capecitabine (ECX and CX) respectively (Table 3). However, both trials were terminated prior to completion due to a greater number of deaths in the patient group receiving rilotumumab as compared to the placebo group [159, 160]. It may be speculated that the case selection, based upon the status of MET protein expression through IHC, may have been responsible for the poor survival outcomes in both studies. Presently, there is a lack of consensus on IHC scoring for MET protein expression. The procedure for MET IHC requires standardization to overcome variability in results from a multitude of factors, such as protocols for staining and tissue processing among others [161]. Efforts to explore alternative biomarker platforms, such as IHC or ISH for HGF expression, may also be considered in future studies.

## FGFR

FGFRs are transmembrane receptor tyrosine kinases. Together with fibroblast growth factors (FGF), they are involved in several biologic processes such as differentiation, angiogenesis, mitogenesis and migration [162, 163]. The FGFR family has four members (FGFR1-4), each bearing several isoforms due to alternative mRNA splicing that allows for diverse ligand binding affinities for each isoform [163–169]. Genetic aberrations in FGFRs have been associated with carcinogenesis in several organ systems.

Numerous studies have highlighted the association between genetic abnormalities in members of the FGFR family and tumorigenesis. FGFR deregulation may be the consequence of either amplification, translocation or a point mutation [170]. Aberrations in FGFRs have been implicated in cancers of the bladder, endometrium, breast and stomach [171–183]. In addition, FGFR translocation and mutation have been associated with multiple myeloma and rhabdomyosarcoma, respectively [184–187]. Thus, therapeutic targeting of FGFR may be beneficial in the above malignancies.

Several phase I and phase II trials are presently underway to evaluate FGFR targeting agents.

The anti-FGFR agents being assessed fall into three categories, namely multikinase/nonselective FGFR inhibitors, selective FGFR inhibitors and monoclonal antibodies that target the FGFR pathway [188]. Although the initial results with FGFR inhibitors are encouraging, the survival benefit may be improved with a robust biomarker platform to enhance case selection.

There are several challenges in the development of a predictive biomarker for anti-FGFR pathway agents. FISH has been used in several studies to ascertain the status of FGFR amplification (Table 4). However, the criteria to determine the status of FGFR by FISH have a significant amount of variation [189–197]. This has led to identification of certain factors that need to be better elucidated prior to defining a standardized protocol for FGFR identification by FISH/ISH. One of the concerns is the heterogeneous or focal pattern of amplification in association with CEN8 polysomy that results in a FGFR/CEN8 ratio < 2.0 even with increased FGFR1 signaling in tumor tissue. Another factor is the unknown influence of genomic heterogeneity in certain tumors such as squamous cell carcinoma of the lung and breast cancer on FGFR expression. It is crucial to create an empiric dataset for defining FGFR cut-offs that are predictive of response to anti-FGFR agents [188]. Alongside the efforts to improve FGFR FISH/ISH, alternative biomarker evaluation strategies are also being explored. Screening assays that can evaluate multiple components of the FGFR pathway for aberrations are under investigation in several studies [188].

**Table 4:** List of clinical trials that assessed FGFR FISH as a biomarker when evaluating FGFR inhibitor based therapy

Drug	Treatment regimen	Biomarker	Tumor histology	Criteria for biomarker positivity	Percent of tumor samples expressing the biomarker	Outcome	Reference
Dovitinib	Dovitinib 500 mg/day, for 5 days followed by 2 days off (28 days cycle)	FGFR FISH/ CSH/SISH	Metastatic breast cancer	≥6 copies of FGFR1 classified as positive for FGFR1 amplification	- 28% (23 of 81 patients) FGFR1+, HR+ - 42% (34 of 81 patients) FGFR1-, HR+ - 27% (22 of 81 patients) FGFR1-, HR- - 2.5% (2 of 81 patients) FGFR1+, HR-; treatment arm terminated prior to completion of study enrollment	Median PFS: - FGFR1+, HR+: 3.6 months - FGFR1-, HR+: 3.5 months - FGFR1-, HR-: 2.1 months SD: - FGFR1+, HR+: 45% (9 of 20 patients) - FGFR1-, HR+: 48% (15 of 31 patients) - FGFR1-, HR-: 25% (4 of 16 patients) PD: - FGFR1+, HR+: 25% (5 of 20 patients) - FGFR1-, HR+: 29% (9 of 31 patients) - FGFR1-, HR-: 31% (5 of 16 patients)	[236]
E3810 (Lucitanib)	Lucitanib 20 mg or 15 mg per day	- FGFR1 FISH; Additional biomarkers (as per study design): - FGFR1 CGH array - FGF3 CGH array	Breast cancer	- FGFR1 FISH: ≥6 copies of FGFR1 per nucleus or FGFR1/CEN8 > 2.2 - FGFR1 CGH array: 8p11-12 amplification log2 ratio > 0.9 - FGF3 CGH array: 11q12-14 amplification log2 ratio > 0.9	- 35% (18 of 51 patients) in expansion cohort - 12 breast cancer patients positive for FGF	10 breast cancer patients were evaluable for response. - PR: 7 patients; 1 additional case exhibited response in bone lesions on PET scan - SD: 1 patient - PD: 2 patients	[193]

Abbreviations: FGFR: Fibroblast growth factor receptor; CISH: Chromogenic *in situ* hybridization; SISH: Silver *in situ* hybridization; FISH, fluorescent *in situ* hybridization; FGFR1: Fibroblast growth factor receptor 1; HR: Hormone receptor; PFS, progression free survival; SD: Stable disease; PD: Progressive disease; PR, partial response; CGH: Comparative genomic hybridization; FGF3: Fibroblast growth factor 3.

## PD-1/PD-L1

Programmed cell death protein, or PD-1, is a receptor molecule expressed on the surface of B cells, T cells and myeloid cells [198, 199]. The primary function of PD-1 is regulation of adaptive immunity. The binding of PD-1 with corresponding ligands (PD-L1/PD-L2) induces inhibition of functioning and proliferation of T cells [199]. PD-1 expression is known to be elevated in exhausted T cells. Pre-clinical studies have shown that the function of exhausted T cells may be partially restored by preventing the interaction of PD-1 with PD-L1 [200–203]. Agents that inhibit the PD-1/PD-L1 interaction have been extensively investigated for potential application in the treatment of cancer.

Several clinical trials have reported a survival benefit associated with the use of PD-1 inhibitory antibodies in various malignancies. The FDA approved the use of PD-1 inhibitors for the treatment of NSCLC (nivolumab and pembrolizumab), melanoma (nivolumab and pembrolizumab), renal cell carcinoma (nivolumab), head and neck cancer (pembrolizumab), Hodgkin’s lymphoma (nivolumab) and bladder cancer (atezolizumab) [34, 204–213]. In addition to the development of PD-1 targeted agents, the identification of predictive biomarkers has also been stressed for improving the selection patients with the highest potential to benefit from this treatment modality.

The expression of PD-L1 has been assiduously evaluated as a predictive biomarker for therapy with anti-PD-1 monoclonal antibodies (Table 5). In light of the successful application of HER2 IHC for trastuzumab based therapy in breast cancer, PD-L1 IHC has been investigated as a predictive biomarker for PD-1 inhibitory based therapy [214]. A phase I trial evaluating nivolumab in melanoma patients defined PD-L1 status as positive if PD-L1 was detected by IHC (Dako IHC, utilizing 28-8 detection antibody) in ≥5% tumor cells. The trial reported that the use of nivolumab was associated with a higher response rate, OS and PFS in PD-L1 IHC positive patients versus those that tested negative for the same [215]. Although fewer in number, PD-L1 negative patients that did respond to nivolumab demonstrated radiological control of disease at par with PD-L1 positive responders that showed almost 75-100% reductions in tumor burden [214–216]. Using the same PD-L1 IHC positivity criteria, a different trial evaluating combination nivolumab plus ipilimumab in advanced melanoma reported a response in 57% of PD-L1 positive and 41% of PD-L1 negative cases [214, 217]. Another trial, again applying the same criteria for PD-L1 IHC status, assessed the efficacy of nivolumab monotherapy in advanced NSCLC. A response rate of 67% was observed in PD-L1 IHC positive cases while those negative for PD-L1 expression on IHC recorded no response with nivolumab therapy [214, 218]. Results obtained for trials evaluating pembrolizumab in melanoma and NSCLC have been largely similar to clinical trials assessing nivolumab in corresponding tumors. A phase I clinical trial assessing the correlation between clinical outcome of melanoma patients receiving pembrolizumab and tumor PD-L1 expression, defined the cut-off as 1% of stained tumor cells for a positive PD-L1 IHC status. The study exhibited a significant improvement of PFS and ORR in patients positive for PD-L1 IHC as compared to those reported as negative [219]. However, nearly 20% of PD-L1 negative patients showed an improvement in PFS with pembrolizumab [214, 219]. Another phase I trial evaluating pembrolizumab in NSCLC defined PD-L1 positivity as having at least 50% of tumor cells expressing PD-L1 on IHC. At 6 months follow-up, the immune related ORR, OS and PFS were found to be considerably higher in the PD-L1 positive versus the PD-L1 negative group [220]. Thus, although clinical evidence indicates that PD-L1 expression may potentially serve as a biomarker, further research is necessary to overcome issues that affect the predictive value of PD-L1 IHC.

**Table 5:** List of clinical trials that assessed PD-L1 IHC as a biomarker when evaluating targeted therapy agents for PD-L1 positive malignancies

Drug	Treatment regimen	Biomarker	Tumor histology	Criteria for biomarker positivity	Percent of tumor samples expressing the biomarker	Outcome	Reference
Atezolizumab	Atezolizumab 1200 mg every 3 weeks	PD-L1 IHC	Urothelial carcinoma	PD-L1 IHC scoring: - IC0: <1% immune cells positive - IC1: ≥1% and <5% immune cells positive - IC2/3: ≥5% immune cells positive	NA	Primary analysis demonstrated significant improvement in objective response rate for each group. Objective response rates: - IC2/3: 27%, 95% CI: 19 to 37, p< 0.0001 - IC1/2/3: 18%, 95% CI: 18 to 34, p= 0.0004 - all patients: 15%, 95% CI: 11 to 20, p= 0.0058	[212]
Nivolumab	Ipilimumab 3 mg/kg; Nivolumab 1 mg/kg, initial 4 doses once every 3 weeks, then once every 2 weeks.	PD-L1 IHC	Melanoma	- ≥5% tumor cells exhibiting PD-L1 staining of any intensity in ≥100 evaluable tumor cells using an automated IHC assay	- 30% (35 of 118 patients) positive for PD-L1	Nivolumab plus Ipilimumab: - PD-L1 positive: 14 of 24 patients with complete and partial response, ORR: 58.3% (95% CI: 36.6 to 77.9) - PD-L1 negative: 31 of 56 patients with complete and partial response, ORR: 55.4% (95% CI: 41.5 to 68.7) Ipilimumab monotherapy: - PD-L1 positive: 2 of 11 patients with complete and partial response, ORR: 18.2% (95% CI: 2.3 to 51.8) - PD-L1 negative: 1 of 27 patients with complete and partial response, ORR: 3.7% (95% CI: 0.1 to 19.0)	[204]
Pembrolizumab	Pembrolizumab 10 mg/kg bi-weekly; ipilimumab 3 mg/kg once every 3 weeks for 4 doses	PD-L1 IHC	Advanced melanoma	- ≥1% tumor cells with membranous staining using a standardized IHC assay	80.5% (671 of 834 patients) positive for PD-L1	2 weeks pembrolizumab therapy versus ipilimumab - PD-L1 positive subgroup: PFS: HR= 0.53, 95% CI: 0.41 to 0.67 OS: HR= 0.55, 95% CI: 0.40 to 0.76 - PD-L1 negative subgroup: PFS: HR= 0.67, 95% CI: 0.41 to 1.11 OS: HR= 0.91, 95% CI: 0.49 to 1.69 3 weeks pembrolizumab therapy versus ipilimumab - PD-L1 positive subgroup: PFS: HR= 0.53, 95% CI: 0.40 to 0.66 OS: HR= 0.58, 95% CI: 0.42 to 0.79 - PD-L1 negative subgroup: PFS: HR= 0.76, 95% CI: 0.47 to 1.24 OS: HR= 1.02, 95% CI: 0.56 to 1.85	[205]
Nivolumab	Concurrent therapy: cohort 1-5 Cohort 1: ipilimumab 3 mg/kg, nivolumab 0.3 mg/kg; Cohort 2a: ipilimumab 1 mg/kg, nivolumab 3 mg/kg; Cohort 3: ipilimumab 3 mg/kg, nivolumab 3 mg/kg; Cohort 4: ipilimumab 3 mg/kg, nivolumab 10 mg/kg; Cohort 5: ipilimumab 10 mg/kg, nivolumab 10 mg/kg Sequential therapy: cohort 6,7 Cohort 6: nivolumab 1 mg/kg; Cohort 7: nivolumab 3 mg/kg	PD-L1 IHC	Advanced melanoma	- ≥5% tumor cells exhibiting PD-L1 staining of any intensity in ≥100 evaluable tumor cells using a standardized automated IHC assay	- 38% (21 of 56 patients) positive for PD-L1	ORR in concurrent therapy cohorts: - PD-L1 positive: 46% (6 of 13 patients) - PD-L1 negative: 41% (9 of 22 patients) ORR in sequential therapy cohorts: - PD-L1 positive: 50% (4 of 8 patients) - PD-L1 negative: 8% (1 of 13 patients)	[216]
Nivolumab	Docetaxel 75 mg/m^2^ once every 3 weeks; nivolumab 3 mg/kg once every 2 weeks	PD-L1 IHC	Squamous-cell NSCLC	- Pre-determined categorization of PD-L1 expression: ≥1%, ≥5% and ≥10% cells exhibiting PD-L1 staining of any intensity in ≥100 evaluable tumor cells using a standardized automated IHC assay (retrospective analysis of pre-treatment specimens)	- 83% (225 of 272 patients) positive for PD-L1	HR for OS according to PD-L1 expression level (nivolumab versus docetaxel therapy): (PD-L1 expression: unstratified HR (95%CI)) - <1%: 0.58 (0.37 to 0.92) - ≥1%: 0.69 (0.45 to 1.05) - <5%: 0.70 (0.47 to 1.02) - ≥5%: 0.53 (0.31 to 0.89) - <10%: 0.70 (0.48 to 1.01) - ≥10%: 0.50 (0.28 to 0.89) - Not quantifiable at baseline: 0.39 (0.19 to 0.82) HR for PFS according to PD-L1 expression level (nivolumab versus docetaxel therapy): (PD-L1 expression: unstratified HR (95%CI)) - <1%: 0.66 (0.43 to 1.00) - ≥1%: 0.67 (0.44 to 1.01) - <5%: 0.75 (0.52 to 1.08) - ≥5%: 0.54 (0.32 to 0.90) - <10%: 0.70 (0.49 to 0.99) - ≥10%: 0.58 (0.33 to 1.02) - Not quantifiable at baseline: 0.45 (0.23 to 0.89) ^*^ The benefit in survival indices was noted irrespective of PD-L1 expression levels. Therefore, PD-L1 expression was not found to have any predictive or prognostic significance in this study.	[237]
Nivolumab	Docetaxel 75 mg/m^2^ once every 3 weeks; nivolumab 3 mg/kg once every 2 weeks	PD-L1 IHC	Non-squamous NSCLC	- Pre-determined categorization of PD-L1 expression: ≥1%, ≥5% and ≥10% cells exhibiting PD-L1 staining of any intensity in ≥100 evaluable tumor cells using an automated IHC assay	- 78% (455 of 582 patients) positive for PD-L1	Treatment with nivolumab by PD-L1 expression interaction P-value (predictive relationship of PD-L1 level for treatment efficacy with nivolumab): - 1% PD-L1 expression level: OS: p= 0.06; PFS: p= 0.02; ORR: p= 0.002 - 5% PD-L1 expression level: OS: p= < 0.001; PFS: p= < 0.001; ORR: p= 0.002 - 10% PD-L1 expression level: OS: p= < 0.001; PFS: p= < 0.001; ORR: p= 0.002 ORR according to PD-L1 expression for nivolumab therapy: (PD-L1 expression: ORR, CI) - <1%: 9%, 95% CI: 5 to 16 - ≥1%: 31%, 95% CI: 23 to 40 - <5%: 10%, 95% CI: 6 to 17 - ≥5%: 36%, 95% CI: 26 to 46 - <10%: 11%, 95% CI: 6 to 17 - ≥10%: 37%, 95% CI: 27 to 48 ORR according to PD-L1 expression for docetaxel therapy: (PD-L1 expression: ORR, CI) - <1%: 15%, 95% CI: 9 to 23 - ≥1%: 12%, 95% CI: 7 to 19 - <5%: 14%, 95% CI: 9 to 21 - ≥5%: 13%, 95% CI: 7 to 22 - <10%: 14%, 95% CI: 9 to 21 - ≥10%: 13%, 95% CI: 6 to 22	[207]
Nivolumab	Nivolumab 0.3 / 2 / 10 mg/kg every 3 weeks	PD-L1 IHC	RCC	- ≥5% tumor cells exhibiting PD-L1 staining using a standardized automated IHC assay. Additionally, cut-off value of ≥1% was also evaluated.	- 64% (107 of 168 patients) positive for PD-L1: 78 patients with <5% PD-L1 expression and 29 with ≥5% expression.	<5% PD-L1 expression: - Median PFS: 2.9 months (95% CI: 2.1 to 4.2) - ORR: 18% (14 of 78 patients, 95% CI: 10.2 to 28.3) - Median OS: 18.2 months (95% CI: 12.7 to 26.0) ≥5% PD-L1 expression: - Median PFS: 4.9 months (95% CI: 1.4 to 7.8) - ORR: 31% (9 of 29 patients, 95% CI: 15.3 to 50.8) - Median OS: not reached ≥1% for PD-L1 expression: - ORR, median PFS and median OS were found to be similar when comparing PD-L1 positive versus negative patients (data unavailable).	[206]
Nivolumab	Nivolumab 0.1 / 0.3 / 1 / 3 / 10 mg/kg once every 2 weeks for up to 96 weeks.	PD-L1 IHC	Advanced melanoma	- ≥5% tumor cells exhibiting PD-L1 staining using a standardized automated IHC assay (retrospective analysis)	- 43.90% (18 of 41 patients) positive for PD-L1	PD-L1 positive patients: - Median OS: not reached - Median PFS: 9.1 months PD-L1 negative patients: - Median OS: 12.5 months - Median PFS: 1.9 months	[214]
Nivolumab	Nivolumab 1 / 3 / 10 mg/kg every 2 weeks (8 weeks cycle) for up to 96 weeks	PD-L1 IHC	Advanced NSCLC	- ≥5% tumor cells exhibiting PD-L1 staining using a standardized automated IHC assay (retrospective analysis)	Not available	PD-L1 positive tumors: - Median OS: 7.8 months (95% CI: 5.6 to 21.7) - Median PFS: 3.6 months (95% CI: 1.8 to 7.5) PD-L1 negative tumors: - Median OS: 10.5 months (95% CI: 5.2 to 21.2) - Median PFS: 1.8 months (95% CI: 1.7 to 2.3)	[238]
Nivolumab	Nivolumab 3 mg/kg once every 2 weeks; docetaxel 75 mg/m^2^ once every 3 weeks	PD-L1 IHC	Advanced/metastatic squamous cell NSCLC	- No specific cut-off value defined as per study design	- Not applicable	OS HR for nivolumab versus docetaxel therapy, according to PD-L1 expression level: (PD-L1 level: HR (95% CI) - ≥1%: 0.69 (0.45 to 1.05) - <1%: 0.58 (0.37 to 0.92) - ≥5%: 0.53 (0.31 to 0.89) - <5%: 0.70 (0.47 to 1.02) - ≥10%: 0.50 (0.28 to 0.89) - <10%: 0.70 (0.48 to 1.01) The tumor PD-L1 status, however, was not found to be of predictive or prognostic value in this particular study.	[229]
Nivolumab	Nivolumab dose range: 0.1 to 10 mg/kg once every 2 weeks	PD-L1 IHC	NSCLC, melanoma, RCC, colorectal cancer, prostate cancer	- ≥5% tumor cells exhibiting PD-L1 staining using a standardized automated IHC assay	- 45% (17 of 38 patients) with melanoma positive for PD-L1 - 49% (31 of 63 patients) with NSCLC positive for PD-L1 - data presently unavailable for other tumors	PD-L1 positive melanoma patients: - Median OS: 21.1 months (95%CI: 9.4 to <not reported>) - Median PFS: 9.1 months (95%CI: 1.8 to <not reported>) - ORR: 44% PD-L1 negative melanoma patients: - Median OS: 12.5 months (95%CI: 8.2 to <not reported>) - Median PFS: 2.0 months (95% CI: 1.8 to 9.3) - ORR: 17% Note: Data presently unavailable for other tumors	[221]
Nivolumab	Sequential escalation of nivolumab dosage: 1, 3, 10 mg/kg, in addition to randomly assigned cohorts with doses ranging from 0.1 mg/kg to 10 mg/kg	PD-L1 IHC	Advanced melanoma, NSCLC, RCC, castration resistant prostate cancer, colorectal cancer	- ≥5% tumor cells exhibiting PD-L1 staining, verified by 2 pathologists	- 59.52% (25 of 42 patients) positive for PD-L1: 18 melanoma, 7 colorectal, 5 RCC, 10 NSCLC and 2 prostate cancer patients	Objective response: - PD-L1 positive patients: 36% (9 of 25 patients) - PD-L1 negative patients: 0% (0 of 17 patients)	[239]
Nivolumab	Nivolumab 1 mg/kg, escalated to 3 mg/kg	Three probe FISH assay: PDL2 (PDCD1LG2), PDL1 (CD274), control centromeric probe	Hodgkin’s lymphoma	- Malignant Reed-Sternberg cells identified and analyzed; Classified as follows: - Amplified: target to control probe ratio of 3:1 - Chromosome 9p polysomy: target to control probe ratio of 1:1 and >2 copies of each probe - Relative copy gain: target to control probe ratio > 1:1 and < 3:1	- 10 tumor samples available for analysis; All positive for PD-L1/PD-L2 alterations	Primary outcomes (survival indices): - RR: 87%, 95% CI: 66 to 97 - Complete response: 17% (4 patients) - Partial response: 70% (16 patients) - Stable disease: 13% (3 patients) - Polysomy 9p: 8 of 10 samples - PD-L1/PD-L2 gain: 6 of 10 samples - PD-L1/PD-L2 amplification: 4 of 10 samples	[211]
Pembrolizumab	Pembrolizumab 10 mg/kg every 2 weeks	PD-L1 IHC	Recurrent/metastatic SCCHN	- a minimum of 1% tumor cells positive for PD-L1 by IHC	-78% (81 of 104 patients) positive for PD-L1	-60 patients positive for PD-L1 received treatment. - 17% patients (10 of 60 patients) reported to have grade 3-4 drug related adverse events - Overall response in all patients: 18% (8 of 45 patients), 95% CI: 8 to 32 - Overall response in HPV positive patients (38%, 23 patients): 25% (4 of 16 patients), 95% CI: 7 to 52 - Overall response in HPV negative patients (62%, 37 patients): 14% (4 of 29 patients), 95% CI: 4 to 32	KEYNOTE- 012 [210]
Pembrolizumab (MK3475)	MK 3475 10 mg/kg every 3 weeks	PD-L1 IHC	NSCLC	- Cut-off value defined by Youden Index from receiver operating characteristics curve, created using irRC assessments	- 29% (9 of 31 patients) with PD-L1 expression score higher than potential cutoff value - 71% (22 of 31 patients) with PD-L1 expression score lower than potential cutoff value	High PD-L1 expression score group: - ORR (irRC assessment): 67% (6 of 9 patients); 95%CI: 30% to 93% - ORR (RECIST): 57% (4 of 7 patients); 95% CI: 18% to 90% - PFS rate (irRC assessment) at 6 months: 67%, median value not reached; 95% CI: 42 to 100% - OS rate (irRC assessment) at 6 months: 89%, median value not reached; 95% CI: 71% to 100% Low PD-L1 expression score group:	[219]
					- According to RECIST criteria: 26% (7 of 27 patients) with high PD-L1 expression and 74% (20 of 27 patients) with low PD-L1 expression	- ORR (irRC assessment): 0% (0 of 22 patients); 95% CI: 0% to 15% - ORR (RECIST): 5% (1 of 20 patients); 95% CI: <not confirmed> - PFS rate (irRC assessment) at 6 months: 11%, median value: 2.1 months; 95% CI: 3% to 40% - OS rate (irRC assessment) at 6 months: 33%, median value: 3.9 months; 95% CI: 18% to 62%	
Pembrolizumab (MK3475)	MK 3475 2 mg/kg every 3 weeks or 10 mg/kg every 2 weeks or 10 mg/kg every 3 weeks.	PD-L1 IHC	Melanoma	- ≥1% tumor cells exhibiting PD-L1 staining	- 77% (55 of 71 patients)	PD-L1 positive patients: - ORR: 51%^ - Median PFS: 12 months^*^ - 1 year OS rate: 84%^**^ PD-L1 negative patients: - ORR: 6%^ - PFS: 3 months^*^ - 1 year OS rate: 69%^**^ ^ORR p= 0.0012 (Fischer exact) ^*^PFS HR: 0.31, 95% CI: 0.16 to 0.61, p=0.0004 (log rank) ^**^ p=0.2146 (log rank)	[218]

Abbreviations: PD-L1, Programmed death ligand-1; IHC, immunohistochemistry; ORR, overall response rate; PFS, progression free survival; OS, overall survival; HR, hazard ratio; NSCLC, non-small cell carcinoma; CI, confidence interval; RCC, renal cell carcinoma; SCCHN, squamous cell carcinoma of the head and neck; irRC, immune related response criteria.

Several preclinical and clinical studies have highlighted multiple issues associated with the use of PD-L1 IHC as a biomarker. There is a lack of consensus on the cut-off values that would define a positive PD-L1 IHC test result [214]. It has been observed that the tumors responsive to PD-1 inhibitor agents have a large variation in PD-L1 IHC expression, ranging from 14% in renal cell carcinoma (RCC), to near 100% in melanoma [214, 221–226]. On the other hand, tumors such as sarcoma and colorectal cancer that are presently believed to be less responsive to PD-1 inhibitor agents have been reported to exhibit PD-L1 IHC expression in a somewhat similar range [214, 226, 227]. This indicates that PD-L1 IHC status may not be the sole criteria to determine response to PD-1 inhibitors. Another concern associated with using PD-L1 IHC is the heterogeneous expression of PD-L1 protein within the tumor microenvironment, in-turn leading to interassay variability [228]. Lastly, the expression of PD-L1 is known to be dynamic. Several studies have noted that treatment with various anti-cancer agents may influence the expression of PD-L1 in tumor cells [229, 230]. Therefore, an appropriate timing for tumor biopsy needs to be established for optimizing patient selection. In order to achieve standardization of PD-L1 IHC for potential application as a predictive biomarker, it is critical to resolve the aforementioned issues associated with the same.

Recently, an automated PD-L1 IHC assay has been conceptualized in an effort to accomplish standardized testing for PD-L1 status [231]. The system utilizes 28-8 antibody for detecting PD-L1 in FFPE samples. This assay has been evaluated for nivolumab therapy in NSCLC specimens. Akin to the criteria used in nivolumab trials, the scoring system for the assay used 1% and 5% cut-off values for PD-L1 positivity. This scoring system was validated by three Clinical Laboratory Improvement Amendment certified labs [231]. The assay demonstrated high precision and reproducibility for PD-L1 status in NSCLC tissue specimens [231]. Further, data from two phase III clinical trials involving advanced NSCLC patients suggest that this assay may be used to identify candidates for nivolumab therapy [232, 233]. Several clinical studies are presently underway to validate the utility of this assay for case selection in nivolumab therapy [231]. Other PD-L1 IHC antibody clones include 22C3 (companion IHC for pembrolizumab), SP142 (companion IHC for atezolizumab) and SP263 (companion IHC for durvalumab). Nuances in PD-L1 IHC cutoff thresholds, tumor versus immune cell staining, and concordance across antibodies remain a central question in the field. Ongoing studies such as BLUEPRINT hope to reconcile differences between antibodies, however future endeavors will require clinical data to determine whether pathologic differences confer differential clinical outcome for patients.

## Alternative biomarkers

Resolute efforts to identify reliable predictive biomarkers have resulted in evaluation of a diverse range of potential biomarker candidates. PCR based detection of single-locus DNA methylation has been gaining focus as a potential biomarker for cancer prognosis, diagnosis and response to various chemotherapy agents [234]. For the purpose of optimizing the utility of PCR in biomarker detection, newer techniques that have evolved from traditional PCR may serve to be promising tools. Such techniques include sensitive melting analysis after real-time methylation-specific PCR (SMART-MSP), methylation-sensitive high-resolution melting PCR (MS-HRM) and methylation-specific fluorescent amplicon generation PCR (MS-FLAG) among others [234]. O6-methylguanine-DNA methyltransferase (MGMT) is among the first DNA methylation biomarker to be identified [235]. Clinical studies demonstrated that MGMT methylation status, detected using methylation-specific PCR, may serve as a predictive biomarker for glioblastoma patients that were to receive temozolomide [236]. In light of these findings, it is reasonable to state that the potential role of DNA methylation PCR as a predictive biomarker merits comprehensive investigation.

A number of studies have documented an association between the intratumoral infiltration of T-cells and clinical outcomes in several malignancies. Preclinical data suggests that intratumoral cytotoxic T-cell infiltration may well be an indicator of prognosis in an exhaustive list of tumor histologies. This includes merkel cell carcinoma, CRC, urothelial carcinoma, anal squamous cell carcinoma and NSCLC among others [237–241]. In addition to the above, numerous studies have noted high lymphocyte infiltrate to be predictive of response to anti-cancer therapy as well. A retrospective analysis of core biopsies from breast cancer patients that received neoadjuvant therapy found that a higher intratumoral lymphocyte infiltration was associated with greater pathological complete response (pCR) rates as compared to tumors without any infiltrate [242]. Similarly, a study investigating CRC reported a statistically significant correlation between the high density of tumor-infiltrating lymphocytes at the invasive margin of CRC metastases in liver and an increase in PFS with chemotherapy [243]. In view of the above, it may be stated that density of tumor-infiltrating T-cells at the invasive margin may potentially serve as a predictive biomarker, pending substantiation of these findings with further evidence.

## Conclusion

**Figure 1:**
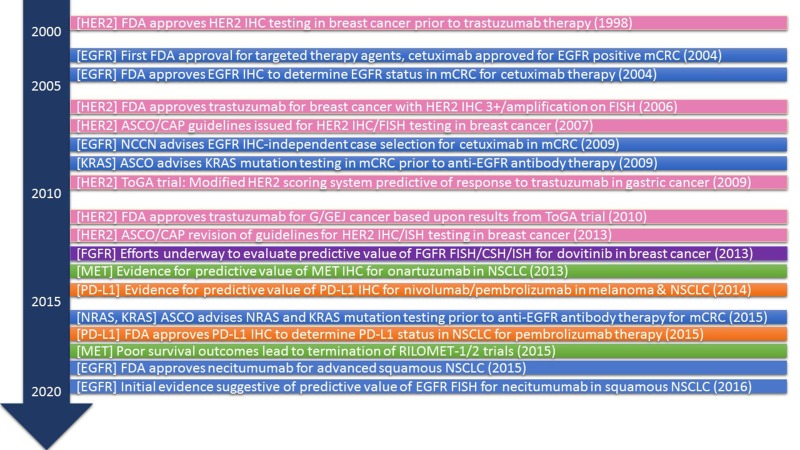
History of predictive biomarkers (IHC and FISH) for targeted therapy in oncology Abbreviations: HER2, human epidermal growth factor receptor-2; FDA, Food and Drug Administration; IHC, immunohistochemistry; EGFR, epithelial growth factor receptor; ASCO, American Society of Clinical Oncology; CAP, College of American Pathologists; FISH, Fluorescent *in situ* hybridization; NCCN, National Comprehensive Cancer Network; mCRC, metastatic colorectal cancer; ToGA, trastuzumab for gastric cancer; G/GEJ, gastric/gastroesophageal cancer; FGFR, fibroblast growth factor receptor; CSH, Chromogenic *in situ* hybridization; ISH, *in situ* hybridization; PD-L1, programmed death ligand-1

The advent of targeted therapy is expected to significantly improve outcomes in patients diagnosed with cancer. The successful application of targeted therapy agents warrants a robust protocol that identifies the presence of the target molecules in the tumor tissues of these patients. In the course of achieving this objective, numerous biomarkers have been developed for several malignancies that are predictive of response to respective targeted therapy (Figure 1).

Initial success in developing predictive biomarkers for trastuzumab therapy in HER2 positive breast cancer has led to new research efforts focused on identifying predictive biomarkers for drugs that target other molecules. These include EGFR IHC/FISH, FGFR FISH, MET IHC and PD-L1 IHC for respective targeted therapy agents in a variety of tumor histologies (Figure 1). However, several challenges have been uncovered in this pursuit.

There are several issues associated with the development and use of predictive biomarkers. One of the key concerns with biomarker testing is the definition of a positive test result. The criteria for positivity for each biomarker is a matter of persistent dispute since a lenient criteria would lead to unnecessary therapy and too strict of a criteria will lead to the exclusion of cases that could possibly benefit from targeted therapy agents. In order to resolve this issue, some clinical studies (evaluating PD-L1 IHC as a possible predictive biomarker) have presented outcomes stratified on the basis of a range of PD-L1 IHC positivity definitions. This approach may be replicated for other biomarkers as well to create a database that would serve a crucial role in defining the most appropriate cut-off for a positive test.

Several concerns associated with the procedure of biomarker testing warrant attention. The traditional methods for testing biomarkers lack uniformity and cannot be precisely replicated each time they are performed. This shortcoming has been partly overcome by the introduction of automated assays, yet a significant amount of operator variability is observed. As with HER2 testing in breast cancer, uniform sets of guidelines should be established for biomarker processing and analysis in order to standardize the testing process. In addition to the above, the reasons for one technique being a superior choice over the other for various biomarkers remain to be unclear [244–246]. As previously noted, FISH appears to be a better option in some while in others, IHC appears to be a better technique to evaluate biomarker status [244, 246]. Studies exploring the differences in mechanics and molecular aspects of these techniques would enhance our understanding of the variables that provide an edge to one technique over the other for a specific target. This knowledge would serve to assist in the selection of an ideal biomarker for a particular target molecule.

The feat of achieving maximum benefit from targeted therapy in cancer patients is currently hindered by numerous inadequacies in biomarker testing. Building upon our experiences thus far, we must apply lessons learned from focused research efforts to continue developing predictive biomarkers for anti-cancer therapy. It is truly essential that the challenges associated with biomarker development be met and resolved to achieve better outcomes in the treatment of cancer.
